# Expression profiles of oviductal mRNAs and lncRNAs in the follicular phase and luteal phase of sheep (*Ovis aries*) with 2 fecundity gene (*FecB*) genotypes

**DOI:** 10.1093/g3journal/jkad270

**Published:** 2023-12-05

**Authors:** Weihao Chen, Zhifeng Li, Rongzhen Zhong, Wei Sun, Mingxing Chu

**Affiliations:** Key Laboratory of Animal Genetics and Breeding and Reproduction of Ministry of Agriculture and Rural Affairs, Institute of Animal Science, Chinese Academy of Agricultural Sciences, Beijing 100193, China; College of Animal Science and Technology, Yangzhou University, Yangzhou 225009, China; Joint International Research Laboratory of Agriculture and Agri-Product Safety of Ministry of Education of China, Yangzhou University, Yangzhou 225009, China; Key Laboratory of Animal Genetics and Breeding and Reproduction of Ministry of Agriculture and Rural Affairs, Institute of Animal Science, Chinese Academy of Agricultural Sciences, Beijing 100193, China; College of Animal Science and Technology, Yangzhou University, Yangzhou 225009, China; Jilin Provincial Key Laboratory of Grassland Farming, Northeast Institute of Geography and Agroecology, Chinese Academy of Sciences, Changchun 130102, China; College of Animal Science and Technology, Yangzhou University, Yangzhou 225009, China; Joint International Research Laboratory of Agriculture and Agri-Product Safety of Ministry of Education of China, Yangzhou University, Yangzhou 225009, China; Key Laboratory of Animal Genetics and Breeding and Reproduction of Ministry of Agriculture and Rural Affairs, Institute of Animal Science, Chinese Academy of Agricultural Sciences, Beijing 100193, China

**Keywords:** sheep, oviduct, lncRNA, mRNA, FecB, follicular phase, luteal phase

## Abstract

*FecB* (also known as *BMPR1B*) is a crucial gene in sheep reproduction, which has a mutation (A746G) that was found to increase the ovulation rate and litter size. The *FecB* mutation is associated with reproductive endocrinology, such mutation can control external estrous characteristics and affect follicle-stimulating hormone during the estrous cycle. Previous researches showed that the *FecB* mutation can regulate the transcriptomic profiles in the reproductive-related tissues including hypothalamus, pituitary, and ovary during the estrous cycle of small-tailed Han (STH) sheep. However, little research has been reported on the correlation between *FecB* mutation and the estrous cycle in STH sheep oviduct. To investigate the coding and noncoding transcriptomic profiles involved in the estrous cycle and *FecB* in the sheep oviduct, RNA sequencing was performed to analyze the transcriptomic profiles of mRNAs and long noncoding RNAs (lncRNAs) in the oviduct during the estrous cycle of STH sheep with mutant (*FecB*^BB^) and wild-type (*FecB*^++^) genotypes. In total, 21,863 lncRNAs and 43,674 mRNAs were screened, the results showed that mRNAs had significantly higher expression levels than the lncRNAs, and the expression levels of these screened transcripts were lower in the follicular phase than they were in the luteal phase. Among them, the oviductal glycoprotein gene (*OVGP1*) had the highest expression level. In the comparison between the follicular and luteal phases, 57 differentially expressed (DE) lncRNAs and 637 DE mRNAs were detected, including *FSTL5* mRNA and LNC_016628 lncRNA. In the comparison between the *FecB*^BB^ and *FecB*^++^ genotypes, 26 DE lncRNAs and 421 DE mRNAs were detected, including *EEF1D* mRNA and LNC_006270 lncRNA. Gene Ontology and Kyoto Encyclopedia of Genes and Genomes functional enrichment analysis indicated that the DE mRNAs were enriched mainly in terms related to reproduction such as the tight junction, SAGA complex, ATP-binding cassette, nestin, and Hippo signaling pathway. The interaction network between DE lncRNAs and DE mRNAs indicated that LNC_018420 may be the key regulator in sheep oviduct. Together, our results can provide novel insights into the oviductal transcriptomic function against a *FecB* mutation background in sheep reproduction.

## Introduction

Lambing traits are considered to be one of the most economically important traits in the sheep industry; however, the molecular mechanisms underlying these traits are still not fully understood. Booroola fecundity (*FecB*), also known as bone morphogenetic protein receptor 1B (*BMPR1B*), is the first gene that was shown to be strongly associated with the fecundity of Booroola sheep and to harbor a mutation (A746G) (Davis *et al.* 2002). This mutation results in an amino acid substitution (Q to R) in the FecB protein and can lead to an additive effect on ovulation number and litter size; 1 copy of the *FecB* mutation can increase the ovulation number by 1.3–1.6 and the litter size by 0.9–1.2, and 2 copies can increase the ovulation number and litter size by 2.73 and 1.1–1.7, respectively (Wang *et al.* 2021). In addition to Booroola sheep, the *FecB* mutation has been identified in Chinese indigenous sheep breeds, it is reported that the litter size of small-tailed Han (STH) sheep carried *FecB* mutation (*FecB*^BB^) were significantly higher (0.92–1.02) than that with wild-type (*FecB*^++^) genotypes, and the *FecB* mutation also had a marked increasing effect on the litter size of Hu sheep, Tan sheep, Luxi Blackhead sheep, etc. Nevertheless, recent observations indicated that ewes with variations in *FecB* could produce singletons (Tian *et al.* 2020). Hence, the molecular mechanisms of *FecB* remain to be determined and warrant further investigation.

Long noncoding RNAs (lncRNAs) are a kind of noncoding RNAs (ncRNAs) with a length of more than 200 nucleotides, which do not encode proteins and were previously referred to as junk RNA (Herman *et al.* 2022). The lncRNAs were proved to have various essential biological roles in, for example, reproduction (He *et al.* 2021), cancers (Peng *et al.* 2017), and muscle development (Baghdadi and Tajbakhsh 2018). In addition, the competitive endogenous RNA (ceRNA) hypothesis demonstrated that lncRNAs can act as microRNA (miRNA) sponges to regulate the expression of target genes involved in various biological activities (Tay *et al.* 2014). The lncRNAs-correlated ceRNA regulatory in female reproduction has been widely studied (Wang and Qin 2019; Sun *et al.* 2021). For example, the lncRNA DANCR was shown to regulate the migration and invasion of trophoblast cells through microRNA-214-5p (Zhang *et al.* 2021), and lncRNA NEAT1 was shown to affect endometrial receptivity by regulating *HOXA10* (Geng *et al.* 2022).

In light of these discoveries, it was assumed that lncRNA plays a similar role in sheep reproduction, and may further be involved in regulating the *FecB* mutation. Sheep reproduction is a dynamic and complicated progress which is regulated by ovarian follicle development, ovulation, and luteinization (Pourlis 2011). As a functional connection between the ovary and the uterus, the oviduct is essential in the preimplantation process of zygotes and embryos (Li and Winuthayanon 2017). Previous research in Rasa Aragonesa sheep has characterized the gene expression during early gestation (Fernandez-Foren *et al.* 2023), and identified subsets of metabolic and reproductive potential candidate genes. However, comparison of the sheep oviductal transcriptomic profiles during the estrous cycle is still lacking, especially noncoding RNA. To investigate the coding and noncoding transcriptomic regulation involved in the estrous cycle and *FecB* in the sheep oviduct, RNA-seq was performed on the oviductal transcriptomic profiles of STH sheep with different *FecB* genotypes. Systematic functional analysis was conducted to obtain an in-depth understanding of the underlying molecular mechanisms and to screen for potential lncRNA or mRNA biomarkers related to sheep estrous cycle.

## Materials and methods

### Sample collection

The experimental STH sheep supplied by Yuncheng Breeding Sheep Farm (Yuncheng, Shandong Province, China) were genotyped in a previous study. Details about the treatment of the experimental sheep, phenotype identification, and initial processing are available in Zhang *et al.* (2019a). All experimental procedures in this study were approved by the Science Research Department of the Institute of Animal Science, Chinese Academy of Agricultural Sciences, Beijing, China (permit number: IAS2019-49).

In brief, all experimental sheep and were kept in a sheltered outdoor paddock and were provided with alfalfa hay and concentrate, with clear water available ad libitum, the TaqMan probe was firstly applied to genotype the sheep population (*n* = 890). Then, six *FecB*^++^ sheep and six *FecB*^BB^ with no significant differences in age, weight, and body size (2.5 ± 0.5 years old; weight 70 ± 5 kg) were randomly selected from STH sheep with the *FecB*^++^ genotype (*n* = 142, wild type, w) and STH sheep with the *FecB*^BB^ genotype (*n* = 78, mutant type, M), respectively.

All selected sheep were processed by estrous synchronization with Controlled Internal Drug Releasing Device (CIDR; progesterone 300 mg; Zoetis Australia Pty. Ltd., NSW, Australia) for 12 days. The six sheep, comprising 3 FecB^++^ sheep and 3 FecB^BB^ sheep, were euthanized within 45–48 h after CIDR removal (follicular phase) by administering a pentobarbital sodium overdose (1.5 mg/kg), the remaining six sheep were euthanized on day 9 after CIDR removal (luteal phase). Finally, the selected sheep were divided into 4 groups, including FecB^++^ sheep in the follicular phase (wF), FecB^++^ sheep in the luteal phase (wL), FecB^BB^ sheep in the follicular phase (MF), and FecB^BB^ sheep in the luteal phase (ML), based on their genotype record and estrous cycle, the detailed information of selected sheep were shown in Supplementary Table 7. The oviduct isthmus tissues were collected and stored at −80°C for RNA extraction.

### RNA extraction and sequencing

Total RNA was extracted from the stored oviduct tissues with TRIzol reagent. An RNA Nano 6000 Assay Kit (Agilent Technologies, Santa Clara, CA, USA) was used to evaluate the quality of the extracted RNA, and the RNA integrity number (RIN) was determined using an Agilent 2100 bioanalyzer (Agilent Technologies, Santa Clara, CA, USA); the threshold was set as RIN ≥ 8.0.

Firstly, mRNAs with poly-A tails were enriched by Oligo (dT) magnetic beads. Then, rRNAs (ribosomal RNAs) were depleted by the Ribo-Zero rRNA Removal Kit (Epicenter, San Antonio, TX, USA) by digesting the total RNA, and rRNA-free residues were cleaned by ethanol precipitation. LncRNA and mRNA libraries were constructed using an NEBNext Ultra RNA Library Prep Kit (NEB, Ipswich, MA, USA) for Illumina according to the manufacturer's recommendations. Then, the cDNA of approximately 250–300 bp was screened with the AMPure XP beads system, the PCR products were purified again with AMPure XP beads to obtain the libraries, Qubit 2.0 was used for accurate quantification, and the Agilent Bioanalyzer 2100 system was used to evaluate library quality and sequenced on a HiSeq 2500 platform (pair-end strategy, 150 bp) by Novogene Technology Co., Ltd. (Beijing, China).

### Read assembly

The raw reads in FASTQ format were filtered using fastp (Chen *et al.* 2018) to remove low-quality reads containing poly-N, adapters, or poly A, T, C, or G sequences. The obtained clean reads were mapped to the *Ovis aries* reference genome (Oar_v3.1) using HISAT2 (Kim *et al.* 2015).

Candidate noncoding RNAs with lengths > 200 nt exon numbers ≥ 2 and lacking a canonical open reading frame were identified as candidate lncRNAs. Cuffcompare (Trapnell *et al.* 2012) was used to screen different types of lncRNAs including lincRNA, intronic lncRNA, and antisense lncRNA. Coding and noncoding RNA transcripts were distinguished using Coding-Non-Coding Index (CNCI, Sun *et al.* 2013), Coding Potential Calculator 2 (CPC2, Kong *et al.* 2007), and Protein Families database (Pfam, El-Gebali *et al.* 2019) software. StringTie (Pertea *et al.* 2015) was used to assemble the mRNA transcripts.

### Differential expression analysis

The fragments per kilobase per million mapped reads (FPKM, Trapnell *et al.* 2010) method was used to estimate the expression levels of lncRNA and mRNA transcripts. The DESeq R package (Wang *et al.* 2010) was used to identify differentially expressed (DE) lncRNA and mRNA transcripts in 4 comparisons. lncRNA and mRNA transcripts were considered to be significantly DE when the Benjamini–Hochberg false discovery rate (FDR, Benjamini and Hochberg 1995) adjusted *P*-value (qvalue) was <0.05 (−log10(qvalue) > 1.3) and |log2(fold change)| ≥ 1.

### Prediction of the target genes of LncRNAs and interaction network of DE LncRNA–mRNA pairs

To evaluate the interaction between the mRNAs and lncRNAs, cis- and trans-target genes of the DE lncRNAs were predicted. Coding genes located 100 kb up-/down-stream of the lncRNAs were considered as cis-target genes. Regarding the trans-target gene prediction, Pearson correlation coefficients were calculated between the mRNA expression levels of genes and the lncRNA transcripts. The genes were considered as trans-target genes of corresponding lncRNAs when the |correlation| was ≥0.95 and adjusted *P*-value was <0.01.

Based on the DE analyses results, the DE lncRNA–DE mRNA pairs were selected from the all lncRNA-target gene pairs and were used to construct an interaction network with the Cytoscape software (Shannon *et al.* 2003).

### Gene ontology (GO) and Kyoto Encyclopedia of Genes and Genomes (KEGG) enrichment analyses

Gene ontology (GO) and Kyoto Encyclopedia of Genes and Genomes (KEGG) enrichment analyses of the DE mRNAs and target genes of the DE lncRNAs were performed using the GOseq R package (Young *et al.* 2010) and KOBAS (KO-Based Annotation System, Xie *et al.* 2011), respectively. GO terms and KEGG pathways were considered to be significantly enriched when *P* < 0.05 (Fisher's exact test with FDR multiple test correction).

### Data validation

RNA extraction, preparation of first strand cDNA, and real-time quantitative polymerase chain reaction (RT-qPCR) conditions were as described in a previous study (Zhang *et al.* 2019a). Briefly, 6 lncRNAs and 6 mRNAs were randomly selected to verify the reliability of the RNA-seq data. The primers (Supplementary Table 1) for the selected lncRNAs, mRNAs, and reference gene *GAPDH* were designed using Primer Premier 5 software. The RT-qPCRs were performed with cDNA in triplicate. The 2^−ΔΔCt^ method (Livak and Schmittgen 2001) was used to calculate relative expression levels. The results are shown as the fold change of relative expression levels (mean ± standard error) using GraphPad Prism 6 software.

## Results

### Overview of sequencing results

Averages of 93,948,897 (MF), 93,872,202 (ML), 94,768,814 (wF), and 99,720,231 (wL) raw reads were obtained from the individual sample of each group. The average mapping rates were 97.62% (MF), 96.05% (ML), 96.85% (wF), and 96.84% (wL), and the average numbers of clean reads were 91,719,898 (MF), 90,158,838 (ML), 91,784,720 (wF), and 96,577,435 (wL). Detailed characteristics of the sequencing results are shown in Supplementary Table 2.

**Fig. 1.**
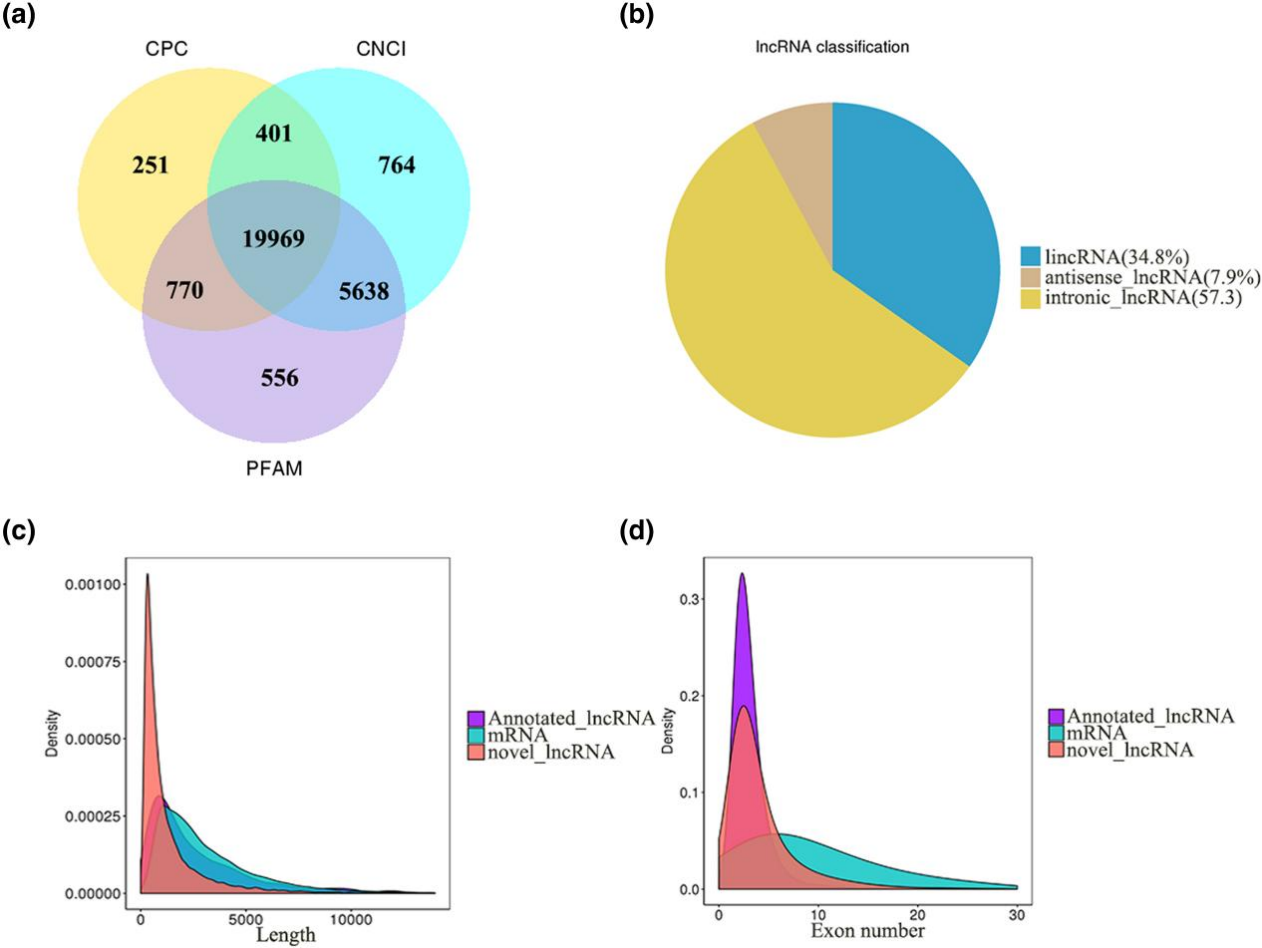
Overview of the RNA sequencing results. a) Classification of coding and noncoding RNAs and identification of long noncoding RNAs (lncRNAs). b) Location of the identified lncRNAs in the genome. c) Length distribution of identified lncRNAs and mRNAs. d) Exon number distribution of identified lncRNAs and mRNAs. CNCI, Coding-Non-Coding Index; CPC, Coding Potential Calculator; PFAM, Protein Families database.

In total, 43,674 mRNAs and 21,863 lncRNAs were identified. Among the lncRNAs, 19,969 were novel lncRNAs (Fig. 1a) and 1,894 were annotated lncRNAs, and 57.3%, 34.8%, and 7.9% of the lncRNAs were intronic lncRNAs, lincRNAs, and antisense lncRNAs, respectively (Fig. 1b). Most of the lncRNAs were 200–3,000 bp long (average 1388 bp) and had 2–8 exons (average 4), whereas most of the mRNAs were 100–5,000 bp long (average 3810) and had an average of 13 exons (Fig. 1c, 1d). Overall, 18,401 cis-target genes of 19,378 corresponding lncRNAs and 8,305 trans-target genes of 11,307 corresponding lncRNAs were predicted.

### Transcriptomic profiling and DE analysis

**Fig. 2.**
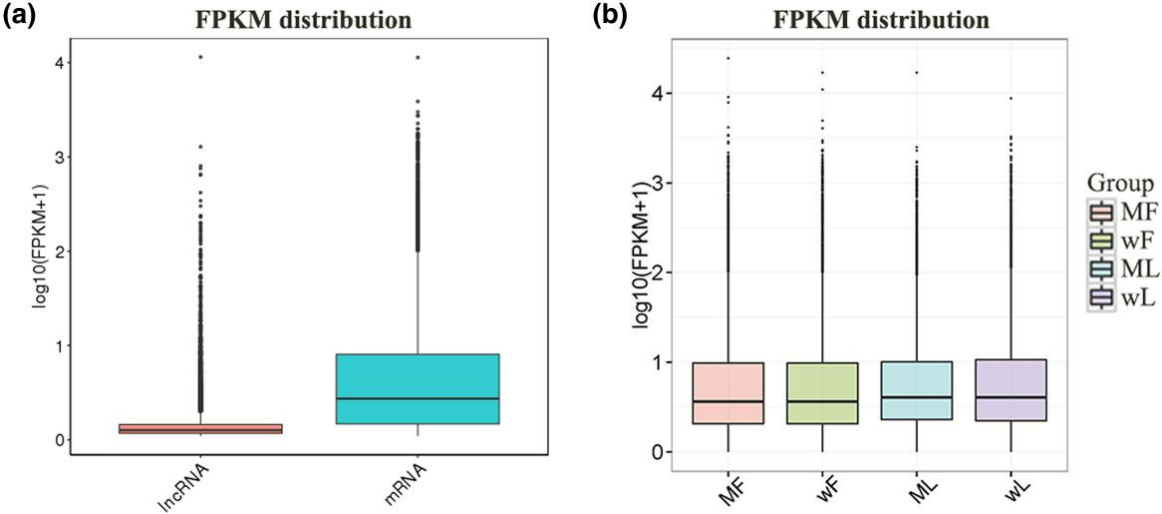
Transcriptomic profiles of the identified lncRNAs and mRNAs. a) Expression levels of the identified lncRNAs and mRNAs. b) Expression levels of the transcripts in different groups. MF, mutant type (*FecB*^++^) STH sheep in follicular phase; wF, wild type (*FecB*^BB^) STH sheep in follicular phase; ML, *FecB*^++^ STH sheep in luteal phase; wL, *FecB*^BB^ STH sheep in luteal phase.

The expression levels of the identified lncRNAs and mRNAs were estimated by calculating the FPKM. The results showed that the mRNAs had significantly higher expression levels than the lncRNAs (Fig. 2a), and that the expression levels of the transcripts were lower in the follicular phase than they were in the luteal phase (Fig. 2b). Details of the DE analyses are provided in Supplementary Table 3.

**Fig. 3.**
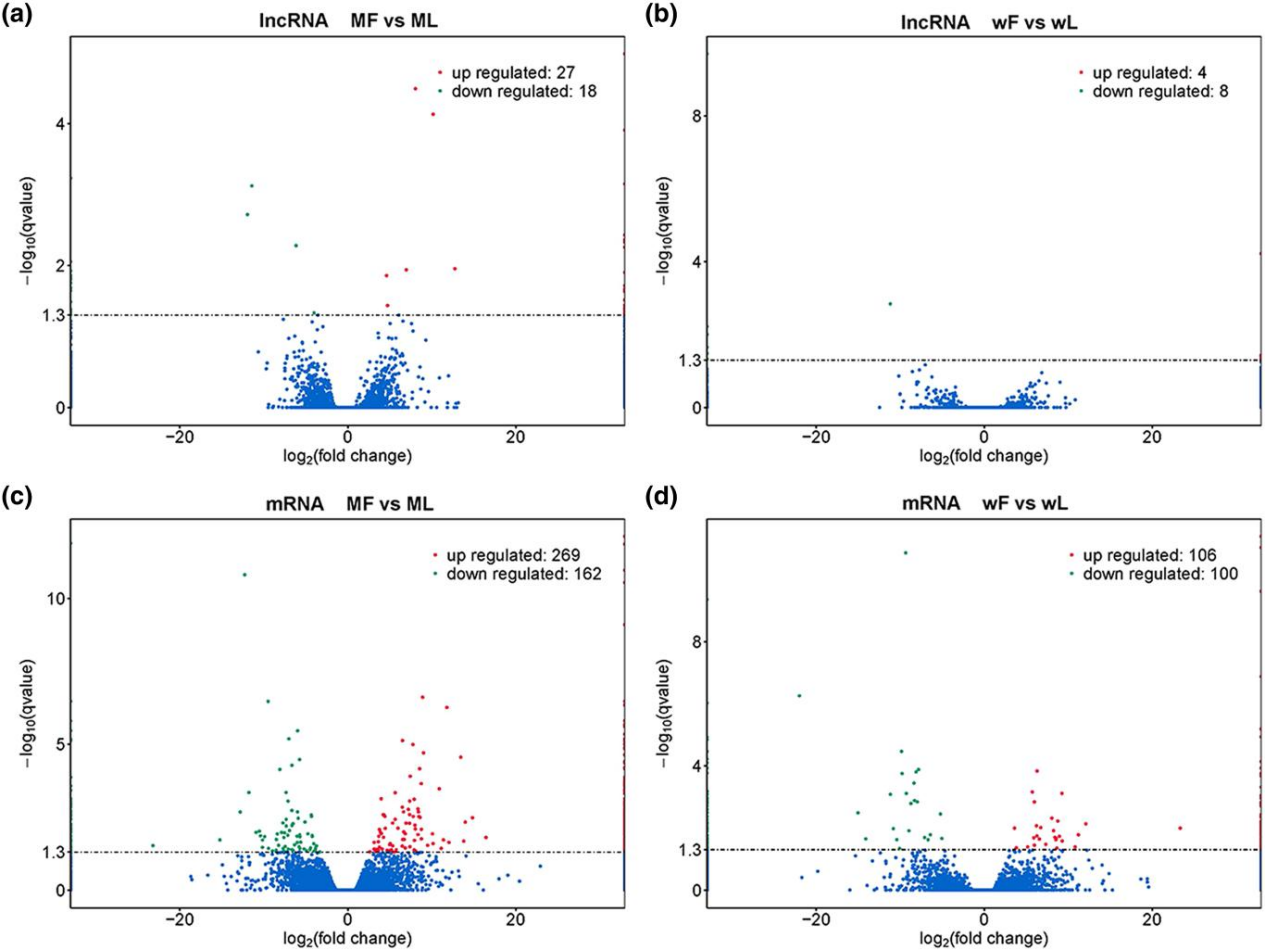
Differentially expressed (DE) lncRNAs and mRNAs in comparisons between the follicular (F) and luteal (L) phases. (a, b) Volcano plots of DE lncRNAs in MF vs ML and wF vs wL. (c, d) Volcano plots of DE mRNAs in MF vs ML and wF vs wL.

In the comparisons between the follicular and luteal phases, 45 (27 up- and 18 down-regulated) and 12 (4 up- and 8 down-regulated) DE lncRNAs were found in MF vs ML (Fig. 3a) and wF vs wL (Fig. 3b), respectively, and 431 (269 up- and 162 down-regulated) and 206 (106 up- and 100 down-regulated) DE mRNAs were found in MF vs ML (Fig. 3c) and wF vs wL (Fig. 3d), respectively.

**Fig. 4.**
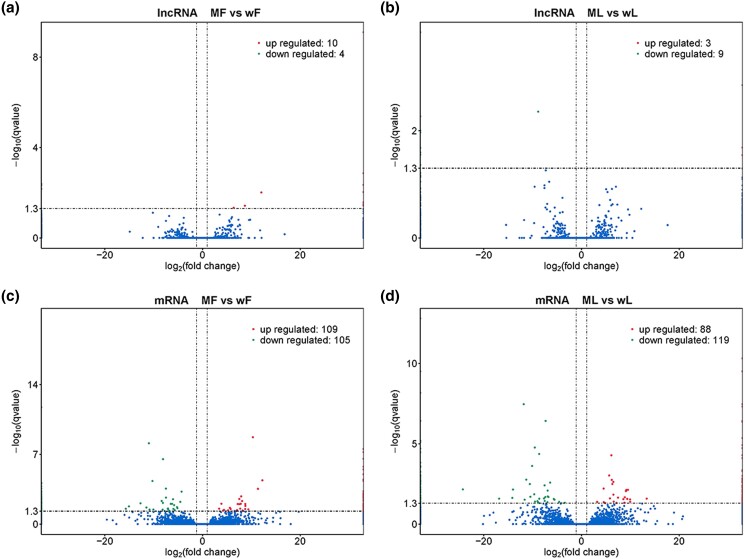
DE lncRNAs and mRNAs in the comparisons between the *FecB*^BB^ and *FecB*^++^ genotypes. (a, b) Volcano plots of DE lncRNAs in MF vs ML and wF vs wL. (c, d) Volcano plots of DE mRNAs in MF vs ML and wF vs wL.

In the comparisons between the *FecB*^BB^ and *FecB*^++^ genotypes, 14 (10 up- and 4 down-regulated) and 12 (3 up- and 9 down-regulated) DE lncRNAs were found in MF vs wF (Fig. 4a) and ML vs wL (Figure.4B), and 214 (109 up- and 105 down-regulated) and 207 (88 up- and 119 down-regulated) DE mRNAs were found in MF vs wF (Fig. 4c) and ML vs wL (Fig. 4d), respectively.

**Fig. 5.**
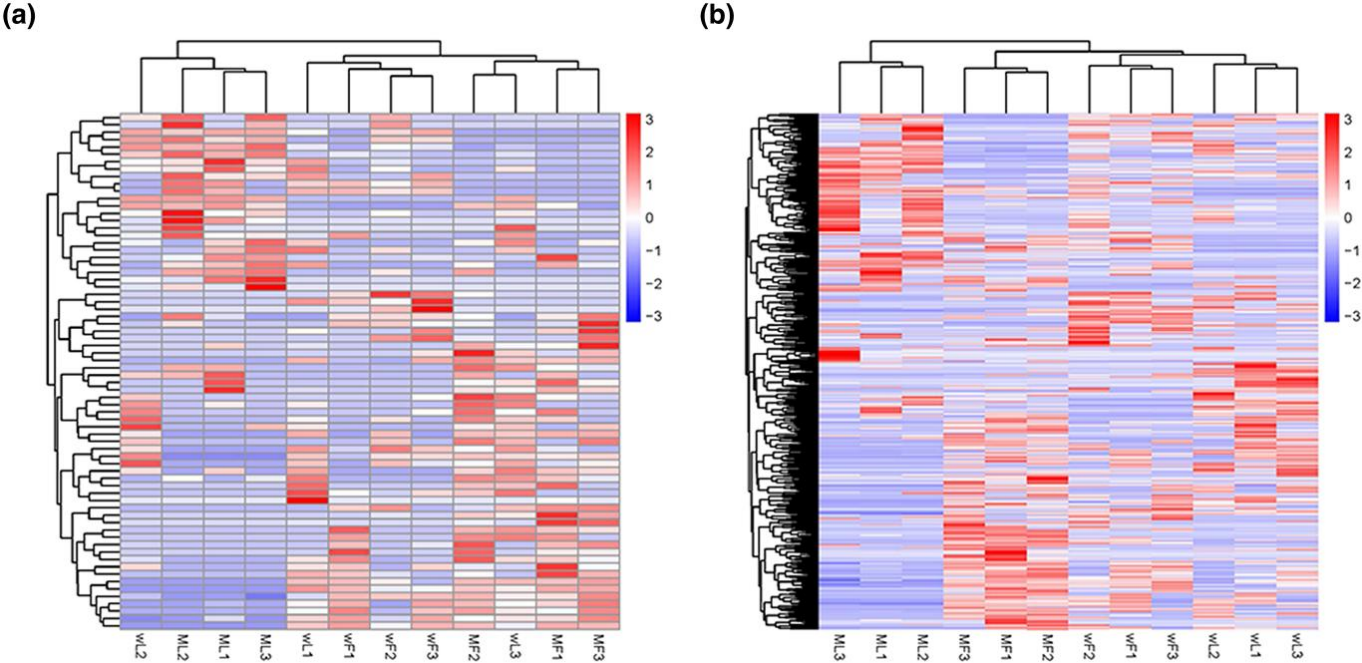
Venn diagrams of the DE lncRNAs and DE mRNAs among different comparison groups. a) DE lncRNAs. b) DE mRNAs.

Venn diagrams of the DE lncRNAs and DE mRNAs among different comparison groups are shown in Fig. 5. LNC_006270 and 4 mRNAs (*C2CD5*, *EPB41L1*, *EML4*, and *VIRMA*) were differentially expressed in all 4 comparisons.

**Fig. 6.**
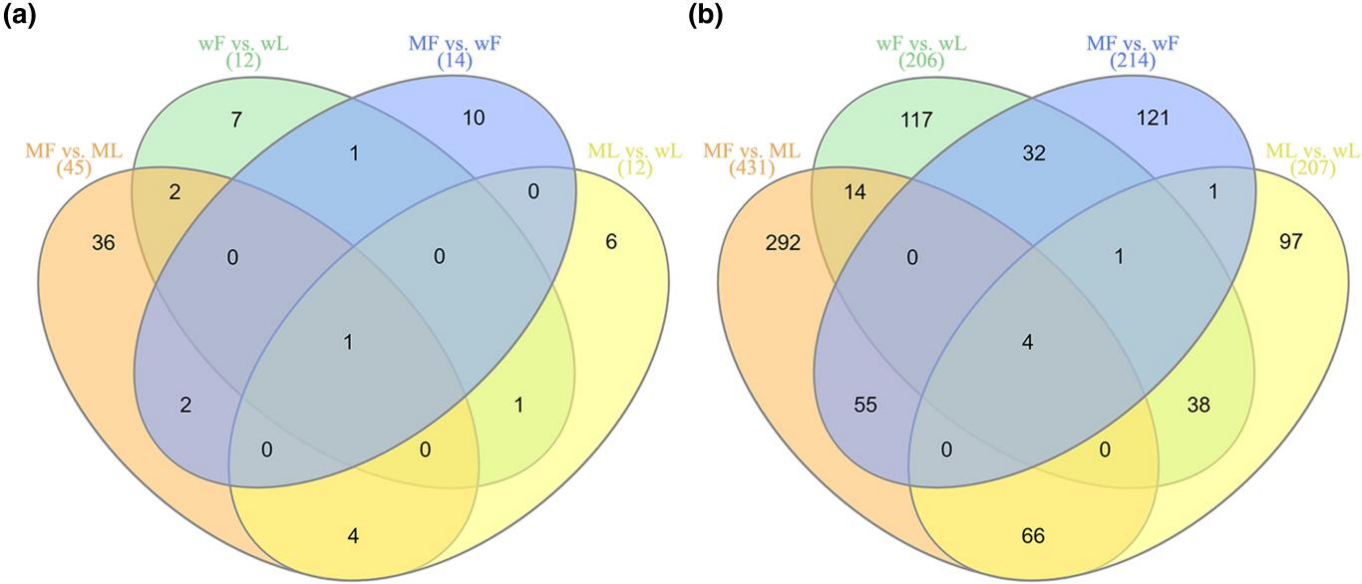
Heatmaps of the transcriptomic profiles of the DE lncRNAs and DE mRNAs. a) DE lncRNAs. b) DE mRNAs.

Heatmaps of the DE lncRNAs and mRNAs showed the differences between the transcriptomic profiles of the MF, ML, wF, and wL groups (Fig. 6). The expression profiles of DE mRNAs between the MF, ML, wF, and wL groups were clearly different.

### GO and KEGG analysis

**Fig. 7.**
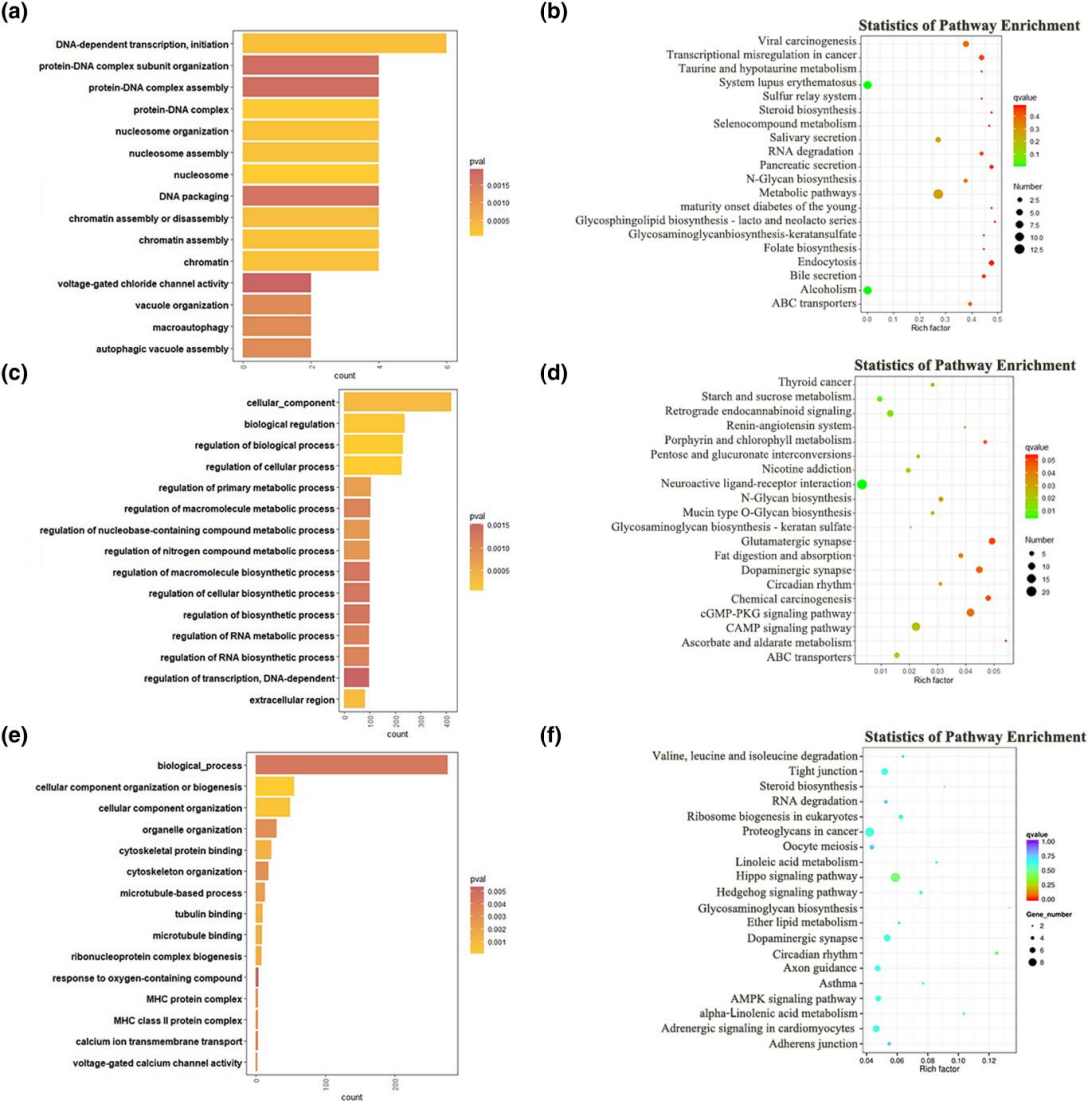
Top enriched GO terms and KEGG pathways of DE mRNAs and target genes of DE lncRNAs in the MF vs ML comparison. a) Top enriched GO terms of cis-target genes of DE lncRNAs. b) Top enriched KEGG pathways of cis-target genes of DE lncRNAs. c) Top enriched GO terms of trans-target genes of DE lncRNAs. d) Top enriched KEGG pathways of trans-target genes of DE lncRNAs. e) Top enriched GO terms of DE mRNAs. f) Top enriched KEGG pathways of DE mRNAs.

**Fig. 8.**
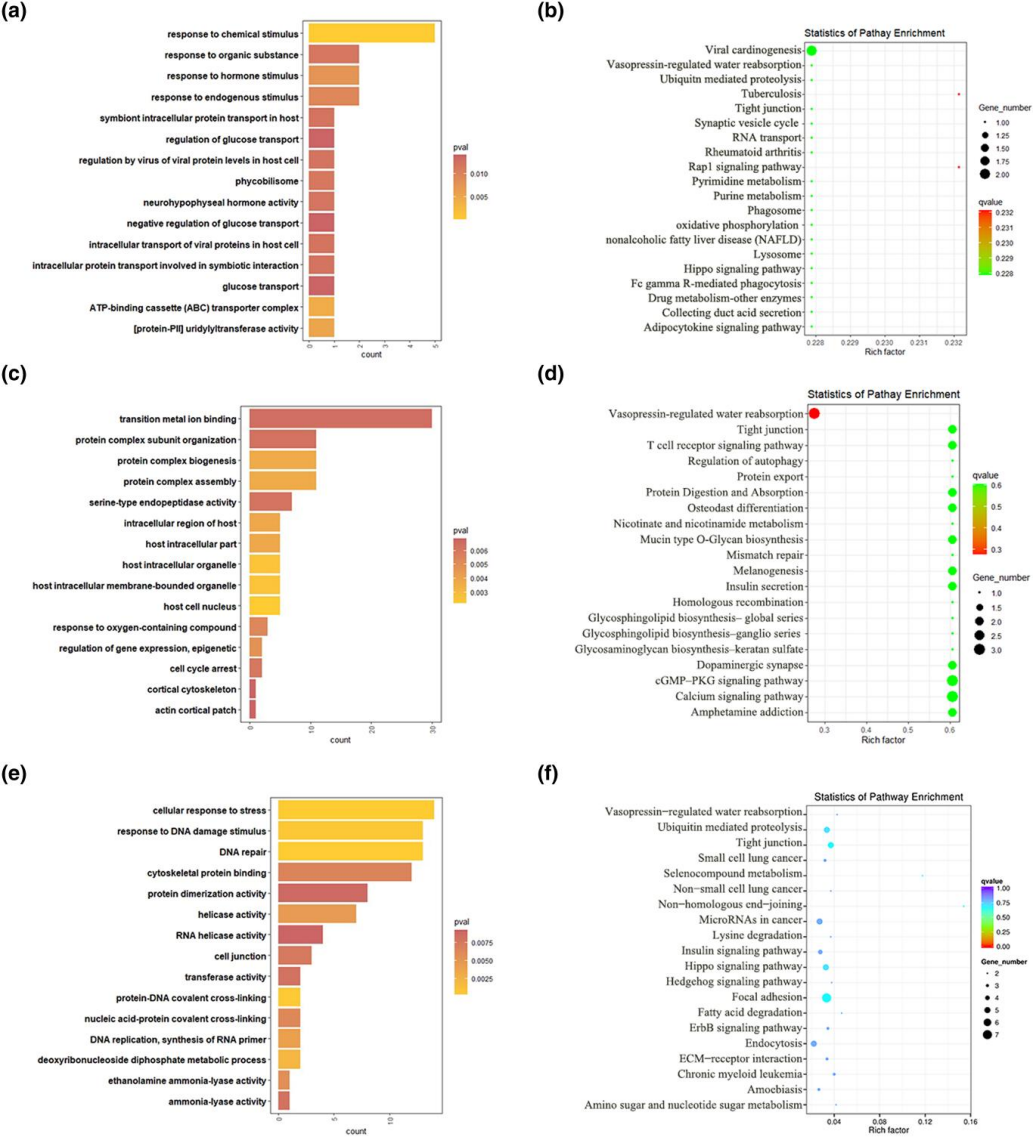
Top enriched GO terms and KEGG pathways of DE mRNAs and target genes of DE lncRNAs in the wF vs wL comparison. a) Top enriched GO terms of cis-target genes of DE lncRNAs. b) Top enriched KEGG pathways of cis-target genes of DE lncRNAs. c) Top enriched GO terms of trans-target genes of DE lncRNAs. d) Top enriched KEGG pathways of trans-target genes of DE lncRNAs. e) Top enriched GO terms of DE mRNAs. f) Top enriched KEGG pathways of DE mRNAs.

**Fig. 9.**
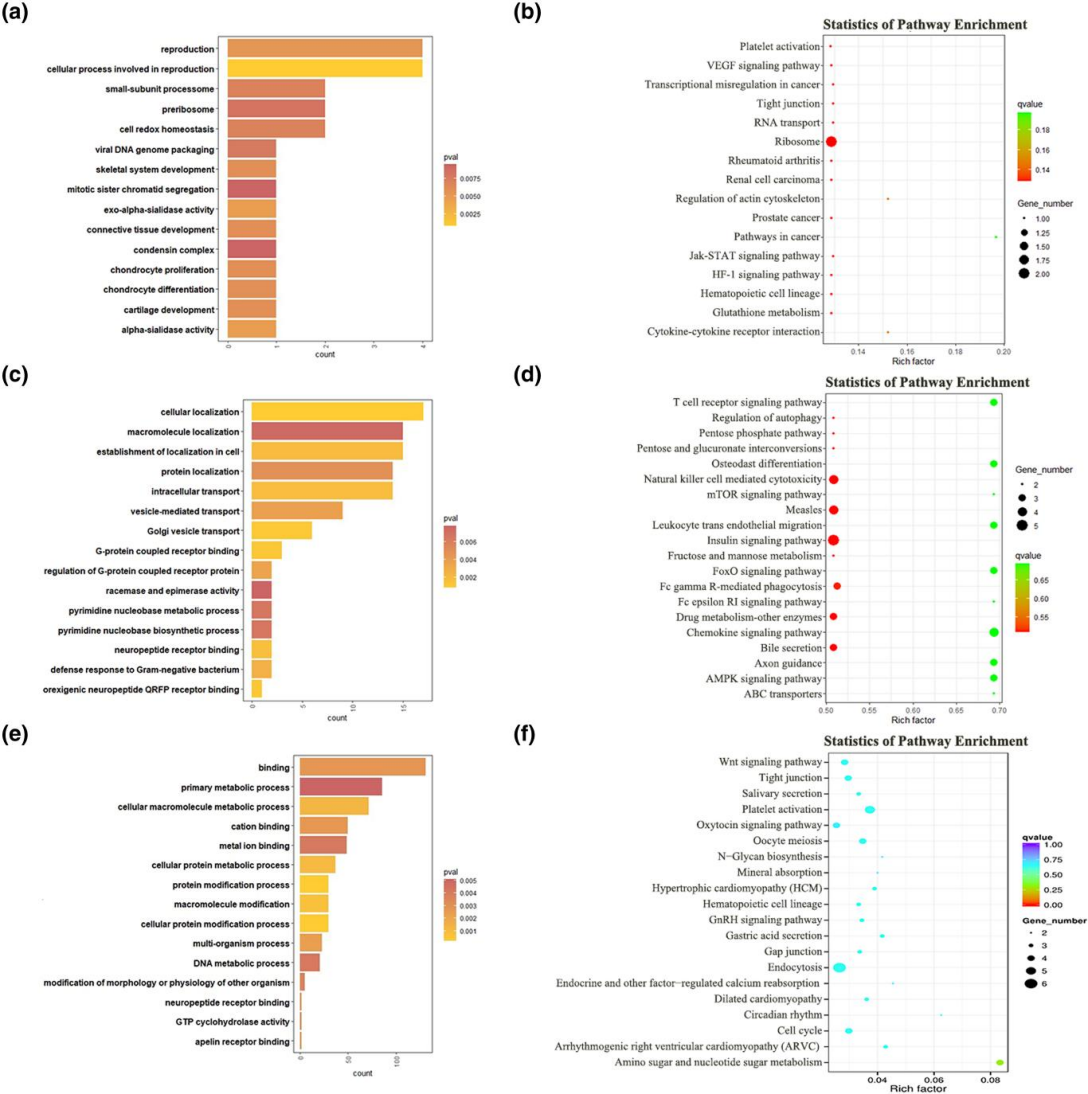
Top enriched GO terms and KEGG pathways of DE mRNAs and target genes of DE lncRNAs in the MF vs wF comparison. a) Top enriched GO terms of cis-target genes of DE lncRNAs. b) Top enriched KEGG pathways of cis-target genes of DE lncRNAs. c) Top enriched GO terms of trans-target genes of DE lncRNAs. d) Top enriched KEGG pathways of trans-target genes of DE lncRNAs. e) Top enriched GO terms of DE mRNAs. f) Top enriched KEGG pathways of DE mRNAs.

**Fig. 10.**
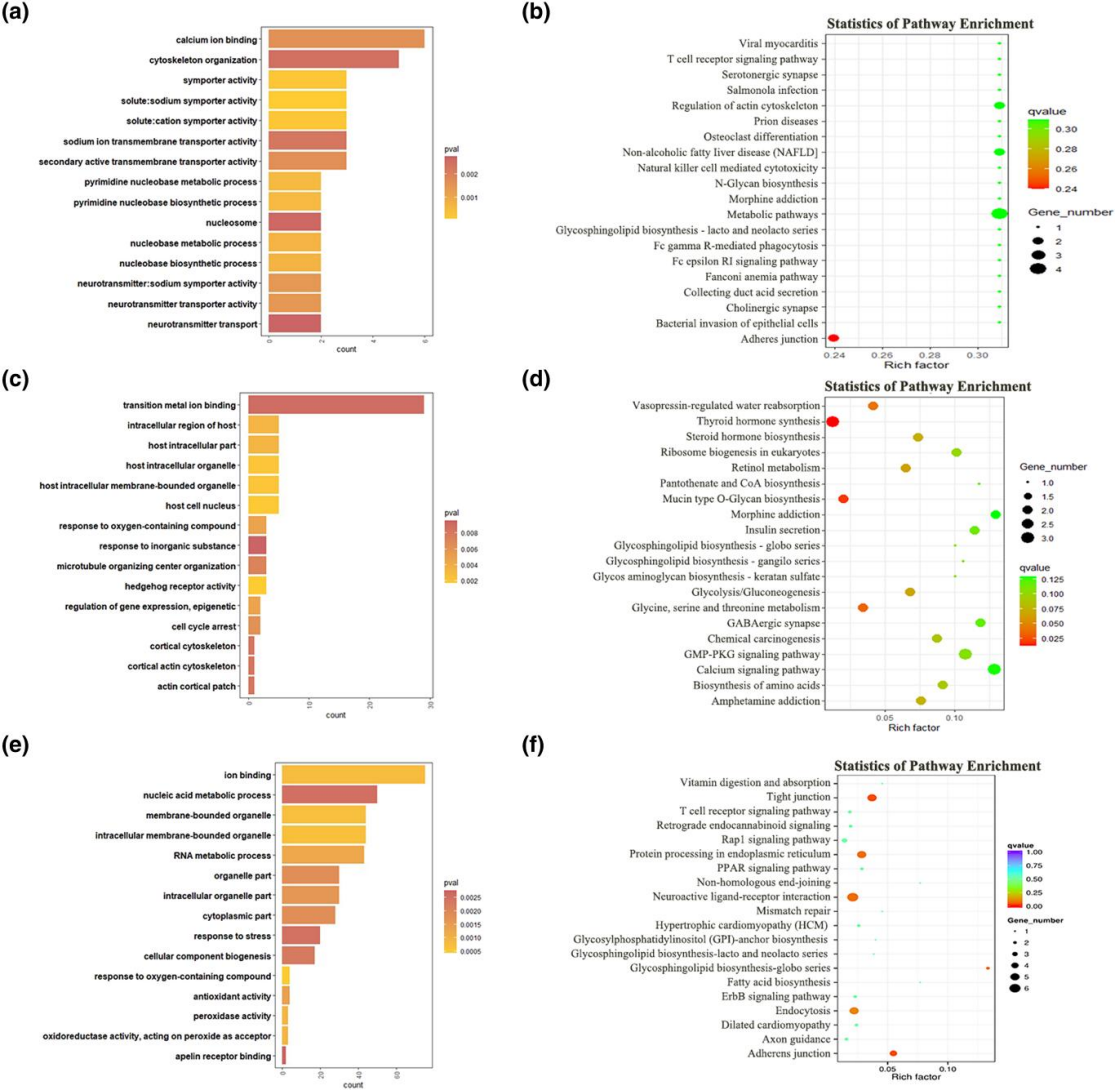
Top enriched GO terms and KEGG pathways of DE mRNAs and target genes of DE lncRNAs in the ML vs wL comparison. a) Top enriched GO terms of cis-target genes of DE lncRNAs. b) Top enriched KEGG pathways of cis-target genes of DE lncRNAs. c) Top enriched GO terms of trans-target genes of DE lncRNAs. d) Top enriched KEGG pathways of trans-target genes of DE lncRNAs. e) Top enriched GO terms of DE mRNAs. f) Top enriched KEGG pathways of DE mRNAs.

To explore the underlying mechanisms of sheep estrous cycle, GO and KEGG analyses were conducted for the target genes of DE lncRNAs and DE mRNAs. The top enriched GO terms and KEGG pathways are shown in Figs. 7–10. Details of the functional enrichment results are provided in Supplementary Tables 4 and 5.

#### GO and KEGG analysis of DE lncRNAs and DE mRNAs in the follicular (F) and luteal (L) phases

In the MF vs ML comparison, cis-target genes of the DE lncRNAs were significantly enriched in 156 GO terms. Among them, the top enriched GO terms were DNA-dependent transcription, protein-DNA complex, and nucleosome. Trans-target genes of the DE lncRNAs were significantly enriched in 156 GO terms. Among them, the top enriched GO terms were regulation of biological process, regulation of cellular process, and biological regulation. The DE mRNAs were significantly enriched in 125 GO terms. Among them, the top enriched GO terms were cellular component organization or biogenesis, cellular component organization, cytoskeletal protein binding (Fig. 7).

In the KEGG analysis, cis-target genes and trans-target genes of DE lncRNAs, and DE mRNAs, were significantly enriched in 7, 19, and 14 KEGG pathways, respectively. Among them, several important pathways related to reproduction were enriched, including the ABC transporters, and AMPK signaling (Fig. 7).

In the wF vs wL comparison, cis-target genes of DE lncRNAs were significantly enriched in 44 GO terms. Among them, the top enriched GO terms response to chemical stimulus, ATP-binding cassette (ABC) transporter complex, and uridylyltransferase activity. Trans-target genes of DE lncRNAs were significantly enriched in 68 GO terms. Among them, the top enriched GO terms were host cell nucleus, host intracellular organelle, and host intracellular membrane-bounded organelle. The DE mRNAs were found significantly enriched in 111 GO terms. Among them, the top enriched GO terms were cellular response to stress, DNA repair and protein-DNA covalent cross-linking (Fig. 8).

In the KEGG analysis, cis-target genes and trans-target genes of DE lncRNAs, and DE mRNAs, were significantly enriched in 4, 3, and 6 KEGG pathways, respectively. Among them, several important pathways related to reproduction were enriched, including the Hippo signaling pathway and metabolism-related pathways (Fig. 8).

#### GO and KEGG analysis of DE lncRNAs and DE mRNAs in the FecB^BB^ (M) and FecB^++^ (w) genotypes

In the MF vs wF comparison, cis-target genes of DE lncRNAs were significantly enriched in 77 GO terms. Among them, the top enriched GO terms were cellular process involved in reproduction, exo-alpha-sialidase activity, and alpha-sialidase activity. Trans-target genes of DE lncRNAs were significantly enriched in 425 GO terms. Among them, the top enriched GO terms were cellular localization, Golgi vesicle transport, and G-protein coupled receptor binding. The DE mRNAs were significantly enriched in 135 GO terms. Among them, the top enriched GO terms were cellular protein modification process, protein modification process and macromolecule modification (Fig. 9). In the KEGG analysis, cis-target genes and trans-target genes of DE lncRNAs, and DE mRNAs, were significantly enriched in 4, 10, and 7 KEGG pathways, respectively. Among them, several important pathways related to reproduction were enriched, including the AMPK signaling pathway and cell cycle (Fig. 9).

In the ML vs wL comparison, cis-target genes of DE lncRNAs were significantly enriched in 118 GO terms. Among them, the top enriched GO terms were solute:sodium symporter activity, symporter activity, and solute:cation symporter activity. Trans-target genes of DE lncRNAs were significantly enriched in 43 GO terms. Among them, the top enriched GO terms were hedgehog receptor activity, host cell nucleus, and host intracellular organelle. The DE mRNAs were significantly enriched in 194 GO terms. Among them, the top enriched GO terms were cellular response to stress, DNA repair, and protein-DNA covalent cross-linking (Fig. 10).

In the KEGG analysis, cis-target genes and trans-target genes of DE lncRNAs, and DE mRNAs, were significantly enriched in 4, 4, and 6 KEGG pathways, respectively. Among them, several important pathways related to reproduction were enriched, including the PPAR signaling and Glycine biosynthesis (Fig. 10).

### Interaction network analysis

**Fig. 11.**
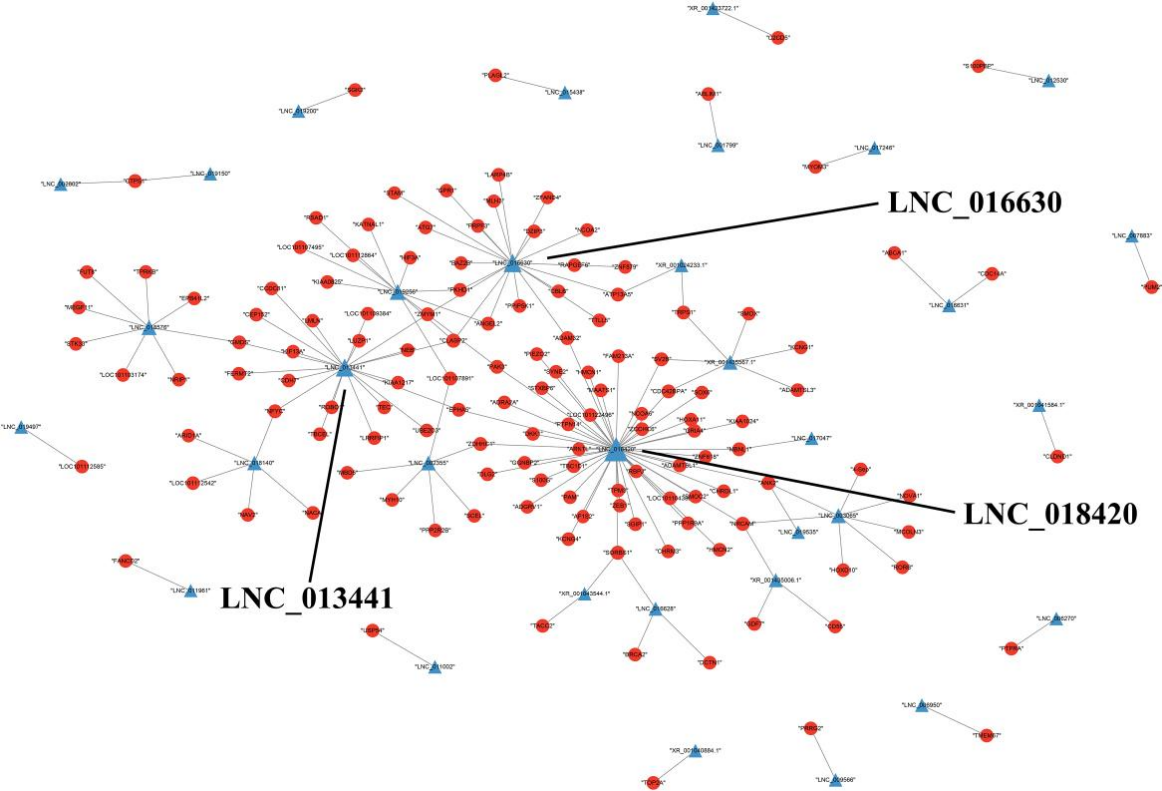
Interaction network constructed using DE mRNAs and corresponding DE lncRNAs.

Overall, 138 DE mRNAs were found to be trans-targets of 33 DE lncRNAs (Supplementary Table 6). Among them, LNC_018420, LNC_016630, and LNC_013441 had the largest number of connections with corresponding DE mRNAs (47, 21, and 20, respectively). The interaction network is shown in Fig. 11 and the network is shown in more detail in Supplementary Fig. 1.

### Sequencing data validation

**Fig. 12.**
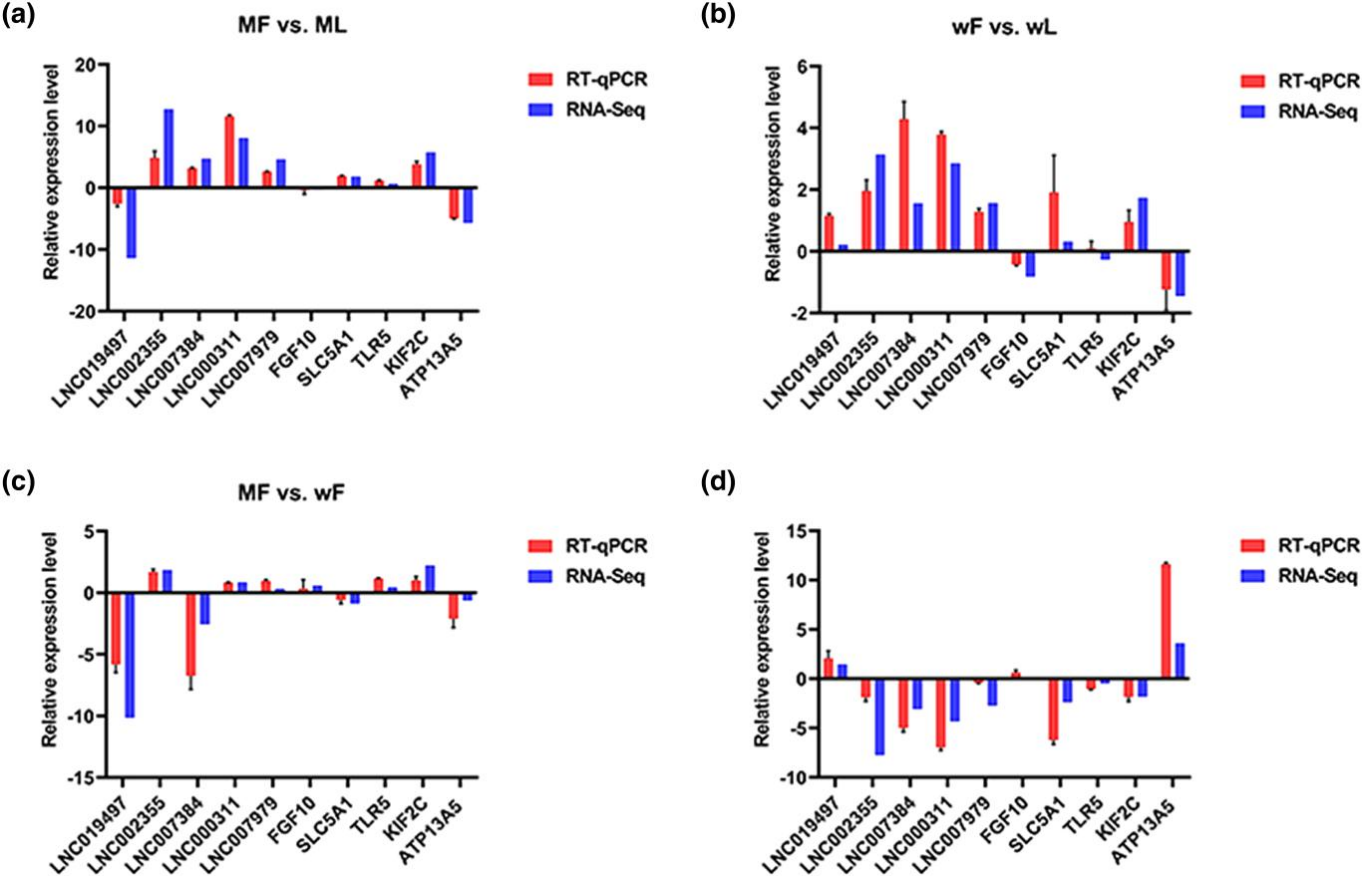
Comparisons of the results of the RNA-seq and RT-qPCR analyses of selected DE lncRNAs and mRNAs. a) MF vs ML. b) wF vs wL. c) MF vs wF. d) ML vs wL.

The relative expression levels of the selected lncRNAs and mRNAs were obtained by RT-qPCR and compared with their expression levels in the RNA-seq data (Fig. 12). Overall, the lncRNAs and mRNAs expression patterns showed similar trends in the RNA-seq and RT-qPCR analyses (R = 0.78 on average), which indicated the reliability and repeatability of our sequencing data.

## Discussion

The ovulation rate of sheep can be genetically controlled at the transcriptional level by several candidate genes, including *FecB* (Davis *et al.* 2002), *BMP15* (Najafabadi *et al.* 2021), and *GDF9* (Chu *et al.* 2011). Previous studies have systemically investigated the transcriptomic profiles of sheep hypothalamus (Zhang *et al.* 2019b), uterus (La *et al.* 2020), and ovary (Li *et al.* 2022) during the estrous cycle, and subsets of coding/noncoding RNAs potentially related to sheep fertility have been discovered. As the connection between ovary and uterus, the oviduct is essential in host fertilization and preimplantation development of the embryo (Li and Winuthayanon 2017); however, little is known about the sheep oviductal transcriptomic profiles during the estrous cycle, especially against a *FecB* mutation background. Hence, RNA-seq of oviduct tissue was performed to investigate how mRNAs and lncRNAs function during the follicular and luteal phases in STH sheep with mutant and wild-type *FecB* genotypes.

### Transcriptomic profiles

In total, 21,863 lncRNAs and 43,674 mRNAs were identified in this study. Overall, the expression levels of the identified transcript were lower in the follicular phase compared with their levels in the luteal phase, and no differences were observed in the expression level of identified transcripts among the different *FecB* genotypes. The present results are consistent with our previous study of miRNAs and circRNAs in sheep oviduct (Li *et al.* 2021). These results indicate that more lncRNAs and mRNAs were activated in the luteal phase than in the follicular phase, and the *FecB* mutations appear to have a relatively smaller effect on the transcriptomic profiles.

Among the annotated mRNAs, *OVGP1* (oviductal glycoprotein 1) had the highest expression level, and among the annotated lncRNAs, LNC_006353 had the highest expression level. Interestingly, the expression levels of *OVGP1* and LNC_006353 were both higher in the follicular phase compared with their levels in the luteal phase. *OVGP1* is oviduct-specific and is closely related to the fertilization rate and embryo development in mammals (Choudhary *et al.* 2019; Zhao *et al.* 2022). In the present study, LNC_006353 was highly expressed in *FecB*^BB^ sheep oviduct during the luteal phase. Besides, the target gene of LNC_006353-*RNF32*, is a type of zinc finger (ZNF) transcription factors containing ring finger motif and reported to regulating the cellular progress of multiple cell types (Wang *et al.* 2016), such as embryonic stem cells (Liu *et al.* 2022). Although little is known about the function of LNC_006353, the high and dynamic expression profile of LNC_006353 suggests that it may be a key regulator in sheep embryo development.

In the comparison between the follicular and luteal phases, 57 DE lncRNAs and 637 DE mRNAs were detected. Similar numbers, 26 DE lncRNAs and 421 DE mRNAs, were detected in the comparison between the *FecB*^BB^ and *FecB*^++^ genotypes. Notably, 4 mRNAs: *C2CD5* (C2 calcium-dependent domain containing 5), *EPB41L1* (erythrocyte membrane protein band 4.1-like 1), *EML4* (echinoderm microtubule associated protein like 4), and *VIRMA* (vir like m6A methyltransferase associated) as well as LNC_006270 were differentially expressed in all 4 comparisons. The influence of these genes on metabolism and cancers has been reported (Han *et al.* 2019; Gavini *et al.* 2020; Jiang *et al.* 2021); however, little is known about their possible association with reproduction. The dynamic expression profiles of these genes detected in this study suggest that they may act as principal regulators of reproduction in sheep oviduct.

In the comparison between the follicular and luteal phases, 18 mRNAs and 3 lncRNAs were differentially expressed in the 2 comparisons; *FSTL5* and LNC_016628 had the highest differential expression (based on fold change). *FSTL5*, which belongs to the follistatin-like protein family, is a glycoprotein that is involved in cell proliferation, migration, differentiation, and embryo development (Zhang *et al.* 2017; Zhang *et al.* 2020a; Khanshour *et al.* 2021). The research in female goat oviduct showed that another crucial member of FSTL family, *FSTL4*, were also found to be significantly differently expressed between high-fecundity goat and low-fecundity goat in the luteal phase (Sun *et al.* 2022), which indicated the essential roles of FSTL family in reproduction. LNC_016628 was specifically expressed in the follicular phase and was predicted to target *MARK3*, a crucial regulator in the lineage commitment of embryonic stem cells (Jin *et al.* 2020). Although the specific roles of *FSTL5* and LNC_016628 in sheep reproduction are still unknown, considering their clearly different expression levels in the follicular and luteal phases, we hypothesized that a regulatory relationship may exist between *FSTL5/*LNC_016628 and embryo development. The specific mechanisms involved require careful characterization in future studies.

The *FecB* mutation has been widely studied in ovary and is a key biomarker in sheep reproduction (Xie *et al.* 2023). During the estrous cycle, the *FecB* mutation can partially inactivate the TGFβ/BMP signaling pathway, suppressing the activity of *GDF5* and *BMP4* gene, and leading to increased ovulation rates (Fabre *et al.* 2003); however, the specific genetically roles of *FecB* in the oviduct have not been well documented. In the comparison between the *FecB*^BB^ and *FecB*^++^ genotypes, the numbers of DE mRNAs and DE lncRNAs were similar to those found in the comparison between the follicular and luteal phases, implying that the *FecB* mutation may have a strong effect on transcription profile in sheep oviduct. Among these genes, LNC_006270 and *EEF1D* had the highest differential expression. LNC_006270 was highly expressed in the oviduct of *FecB*^BB^ sheep, and also targeted *MARK3*, which may be involved in embryo development. *EEF1D* was highly expressed in the oviduct of *FecB*^++^ genotype. Previous embryonic expression studies showed that *EEF1D* was highly expressed in the embryonic stage of mouse (Zhang *et al.* 2020b) and sheep (Han *et al.* 2022), however, the specific role of *EEF1D* in mammal embryonic development is still unclear. In sea urchins, *EEF1D* was revealed highly expressed in unfertilized eggs and early embryos, and then its expression dropped rapidly during the rapid cleavage and blastocyst stages of embryonic development (Delalande *et al.* 1998). Together, these results indicate that the up-regulation of *EEF1D* may have a negative effect on embryonic development in sheep, and the expression of *EEF1D* can be regulated by the *FecB* mutation.

### Functional analysis

In the comparison between the follicular and luteal phases, most of the enriched GO terms were closely related to reproduction-related metabolic processes, such as the SAGA complex and ATP-binding cassette. Regulation of gene expression during embryo development requires cooperation between histone-modifying enzymes and transcription factors such as Gcn5 and PCAF, which together form a multi-subunit complex called SAGA (Bardot *et al.* 2017). ABC transporters shuttle a wide variety of substrates and take part in embryonic development in direct or indirect ways (Joshi *et al.* 2016). Zou *et al.* (2020) found that ABC transporters were enriched in ovarian follicles of uniparous and multiple goats by comprehensive transcriptomic research. Our findings indicated that the SAGA complex and ABC transporters may be key factors in sheep embryo development. In the KEGG analysis, the Hippo signaling pathway was enriched in the follicular and luteal phase comparisons. The Hippo signaling pathway is a key driver in embryo development (Beyer *et al.* 2013; Yildirim *et al.* 2021), and differential Hippo activity has been shown to be highly correlated with inner cell mass and the trophectoderm, the first 2 segregate during early embryo development in mammals (Manzanares and Rodriguez 2013; Anani *et al.* 2014). We speculated that the Hippo signaling pathway influenced reproduction by regulating early embryo development in sheep oviduct.

In the comparisons between the *FecB*^BB^ and *FecB*^++^ genotypes, the enriched GO term: cellular processes involved in reproduction (GO:0048610) and reproduction (GO:0000003) were noted because of their close relationship with embryonic development. The mRNA *NES* (nestin) was enriched in these GO terms, and as the key regulator in the neural response, most nestin-positive cells have been found strongly positive for vimentin (Jung *et al.* 2020), a differentiation marker in preimplantation embryo (Viebahn *et al.* 1988; Tolstonog *et al.* 2005). In the KEGG analysis, most of the enriched pathways were closely related to metabolic pathways, which is consistent with a previous RNA-seq study that found that several metabolic pathways were enriched in the hypothalamus of sheep with different *FecB* genotypes, such as N-glycan biosynthesis, GnRH, and fatty acid biosynthesis (Zhang *et al.* 2019a). Interestingly, as the host signaling pathway of *FecB*, TGFβ/BMP was not enriched in both comparisons. Considering the molecular regulation of the *FecB* mutant, we hypothesized that the *FecB* mutant may have a house-keeping type functional role in the basic metabolic processes of non-ovary tissues in sheep.

In the present research, tight junction and cell junction were enriched in most of the comparisons., even in the comparisons between the *FecB*^BB^ and *FecB*^++^ genotypes. Similarly, the oviductal transcriptome study in *Ascaris lumbricoides* (Phuphisut *et al.* 2022) and goat (Sun *et al.* 2022) also demonstrated that the DE genes were functionally enriched in tight junction. The tight junction in oviduct epithelial cells serves as an essential component of the differentiated epithelial cell required for polarized transport and intercellular integrity and signaling. Regulating a wide range of early embryo development-related processes, which include cell–cell interactions, protein kinase C signaling, gap junctional communication, Na^+^/^K+^-ATPase and cellular energy status (Eckert and Fleming 2008), taken together, the present results further verified the close interlinked relationships between tight junction and sheep reproduction during estrous cycle. It's worth noting that the tight junction term was also enriched in the comparisons between the *FecB*^BB^ and *FecB*^++^ genotypes, considering the function of the *FecB* mutant in reproductive endocrinology, we conclude that the *FecB* mutant may also have some potential effect on the sheep oviductal epithelial cell tight junction and estrous characteristics, and eventually affecting estrus characteristics.

### Interaction network of DE genes

To demonstrate the interaction between DE lncRNAs and corresponding DE mRNAs, we constructed an interaction network. In total, 137 target DE mRNAs of 33 DE lncRNAs were included in the network; LNC_018420 (47) and LNC_016630 (21) were the most highly connected lncRNAs. Among the target genes of LNC_018420 and LNC_016630, some, such as *ZNF* and *ADAMT* genes, were enriched in GO terms and KEGG pathways related to embryo development. ZNF proteins are the largest superfamily of transcript regulators in mammals, and numerous *ZNF* genes have been identified as important biomarkers in the development and differentiation of the nervous system, and are known to interact with multiple regulators in embryonic development (Nakamura *et al.* 2004; Senft and Macfarlan 2021). *ADAMT* proteins have platelet-associated activity, and have been suggested to affect endometrial receptivity in rats, resulting in implantation failure of the embryo (Cai *et al.* 2019). Hence, there is a high probability that LNC_018420 and LNC_016630 are the key regulators in embryo development, and their up- or down-regulation may exert different functions on sheep reproduction.

### Limitations of the study

It is cautionary to mention the limitations of our study, regarding the sample size and sheep breed, and sample collection. The small number of animals (3 for each group) could explain why small subsets of DE transcripts were identified in some comparisons, especially lncRNA. The accuracy results could be achieved by conducting the RNA-seq analyses at high replication levels. Besides, although the *FecB* has been detected in diverse sheep breeds, the effect of FecB mutation may differ across different breeds, for example, in STH sheep, 2 copies of the FecB mutation can increase the litter size of STH by 0.9–1.02, which is relatively lower than their effects on the Booroola sheep (1.1–1.7), hence, the results would potentially be different if the study was performed on oviduct tissue from different sheep breeds. Regarding the sample collection, only sheep of follicular and luteal phases were selected in the present study, the estrous change of sheep oviduct needs to be further investigated considering other phases such as the following follicular phase and diestrous phase. Finally, the present RNA-seq analyses are descriptive, although our results suggest possible reproductive functions of some coding or noncoding transcripts, their specific roles need to be further validated with functional experiments.

## Conclusions

The present oviductal transcriptome analysis reveals the expression profiles of the mRNAs and lncRNAs in sheep with 2 *FecB* genotypes during estrous cycle. In the oviduct of STH sheep, mRNAs and lncRNAs associated with fertilization and early embryo development were significantly enriched during the estrous cycle, such as *OVGP1*, *FSTL5*, LNC_006353, and LNC_018420. Our findings can provide an important reference dataset to study the reproductive function of sheep oviduct and offer subsets candidate genes and lncRNAs linked to oviductal function during estrous cycle in sheep.

## Supplementary Material

jkad270_Supplementary_Data

## Data Availability

The datasets generated in this study have been deposited in the NCBI Gene Expression Omnibus (GEO) database under accession number GSE247022 (https://www.ncbi.nlm.nih.gov). Supplementary Table 1: primers used for the RT-qPCRs and sequences of the lncRNAs selected for sequencing validation; Supplementary Table 2: details of the sequencing data; Supplementary Table 3: details of the differentially expressed mRNAs and lncRNAs in 4 comparisons; Supplementary Table 4: details of the Gene Ontology (GO) enrichment analysis results; Supplementary Table 5: details of Kyoto Encyclopedia of Genes and Genomes (KEGG) enrichment analysis results; Supplementary Table 6: detailed interaction network of differentially expressed mRNA–lncRNA pairs; Supplementary Table 7: detailed information of selected sheep for RNA-seq. Supplementary Fig. 1: high-resolution interaction network of differentially expressed mRNA–lncRNA pairs. Supplemental material available at G3 online.
