# Supplementary figures and images for: Expression profiles of oviductal mRNAs and lncRNAs in the follicular phase and luteal phase of sheep (Ovis aries) with 2 fecundity gene (FecB) genotypes
Source: G3 (Bethesda). 2023 Dec 5;14(1):jkad270. doi: 10.1093/g3journal/jkad270 (PMC10755197; doi:10.1093/g3journal/jkad270)

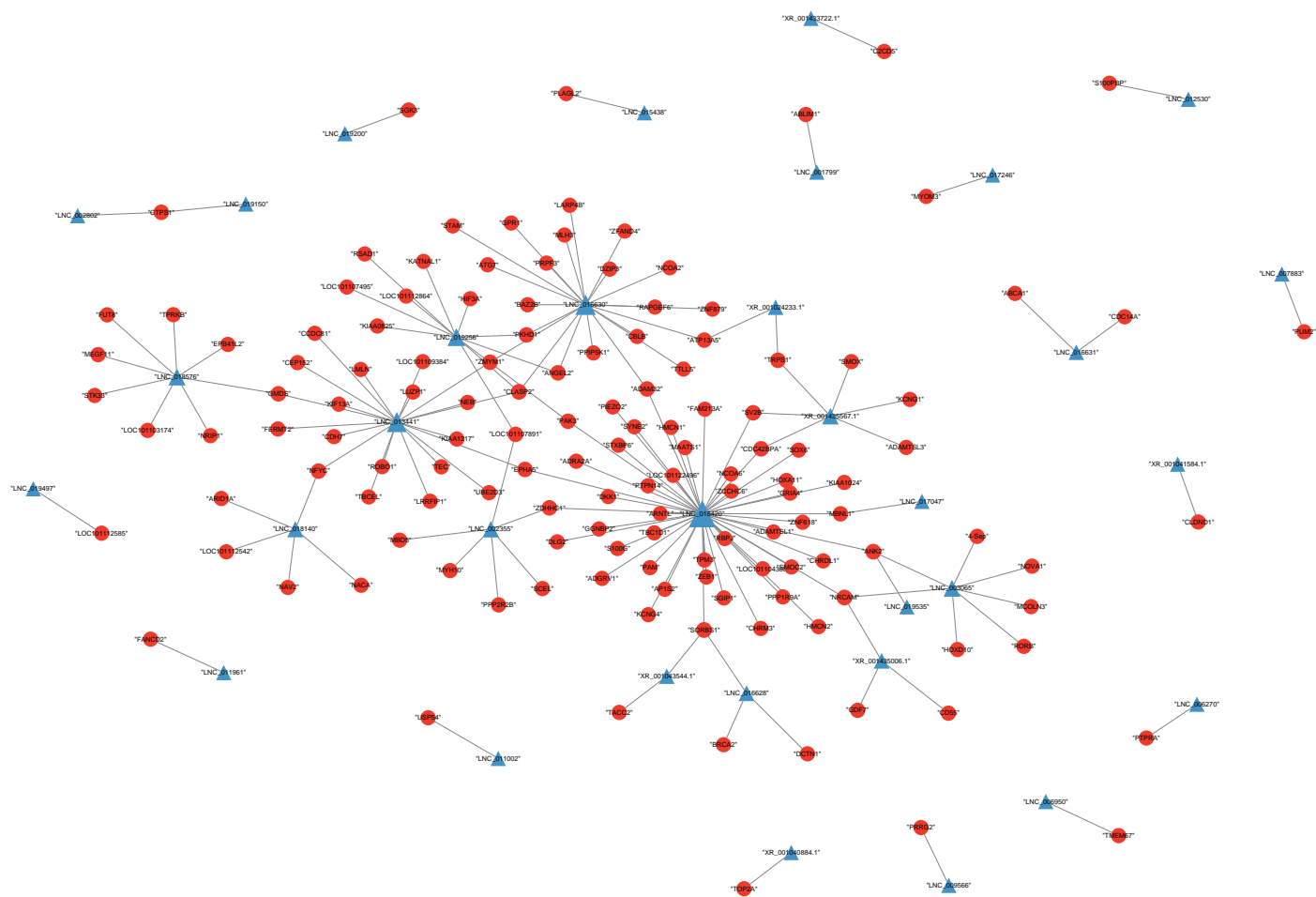

Supplement: jkad270_Supplementary_Data [file jkad270_supplementary_data.zip › Supplementary Figure S1.pdf]
